# Nudge for (the Public) Good: How Defaults Can Affect Cooperation

**DOI:** 10.1371/journal.pone.0145488

**Published:** 2015-12-30

**Authors:** Toke R. Fosgaard, Marco Piovesan

**Affiliations:** 1 University of Copenhagen, Department of Food and Resource Economics, Rolighedsvej 23, 1958 Frederiksberg C, Denmark; 2 University of Copenhagen, Department of Economics, Øster Farimagsgade 5, building 26, 1353 Copenhagen K., Denmark; University of Reading, UNITED KINGDOM

## Abstract

In this paper we test the effect of non-binding defaults on the level of contribution to a public good. We manipulate the default numbers appearing on the decision screen to *nudge* subjects toward a *free-rider strategy* or a *perfect conditional cooperator strategy*. Our results show that the vast majority of our subjects did not adopt the default numbers, but their stated strategy was affected by the default. Moreover, we find that our manipulation spilled over to a subsequent repeated public goods game where default was not manipulated. Here we found that subjects who previously saw the free rider default were significantly less cooperative than those who saw the perfect conditional cooperator default.

## Introduction

Cooperation is an essential aspect of life, from workplace collaboration to environmental conservation, from political participation to international relations [1–3]. To study cooperation, social scientists have used various social dilemma experiments, in particular the public goods game [4–6]. In this game, N subjects secretly choose how many of their private tokens to put into a public pot, which are then multiplied by a factor “a” (1> a > 1/N) with the resulting public good payoff being evenly divided among the players. The dilemma arises since the Nash equilibrium in this game is a zero contribution, but the social optimum is full contribution. The experimental evidence collected in the last three decades has shown that subjects, contrary to their self-interest, often do contribute positive amounts [7,8].

Social scientists interested in the evolution and sustainability of cooperation have looked at the repeated version of the public goods game [9]. In this game, we usually observe that contributions start from 40–60% of the initial endowment, but then decline quickly with repetition [8]. The most convincing explanation for these results is that subjects are heterogeneous in their preferences and beliefs and that selfish and altruistic subjects coexist with *conditional cooperators*. [10]. To categorize preferences for cooperation, Fischbacher and co-authors [11] developed a “strategy version” of the public goods game (the strategy game hereafter). In this game, subjects have to state their desired contributions for all possible (average) contributions of the other group members. The analysis of these “strategies” confirms the coexistence of free riders and conditional cooperators. Moreover, the quick decline in contributions observed in the repeated version of the public good game has been attributed to subjects’ average preference for conditional contributions below the contributions of others[10].

In this paper we propose a mechanism–namely priming through default strategies–to influence contributions both in a strategy version of the public goods game and in a (subsequent) repeated public good game. The previous literature has proposed several mechanisms that can foster cooperation. In particular, researchers have tested and proven the efficacy of: decentralized institutions such as communication [12], framing [13,14] punishment [15], rewards[5]; centralized institutions such as taxation [16] and competition [17]; and endogenous formation of institutions[18].

Our approach is different and in particular we use a subtle manipulation to “nudge” subjects toward specific cooperation strategies (free riding or perfect conditional cooperation). In more detail, we manipulate the default numbers that appear on the screen with the elicitation of the cooperation strategy within the strategy version of the public goods game. In our Free-Rider Treatment, the default numbers correspond to a free riding strategy (FR), whereas in the Perfect Conditional Cooperator Treatment (PCC), the default numbers correspond to a perfect conditional cooperator strategy (one matches the average contribution of others). As a benchmark, we also have a No Default Treatment (ND) where the cell to be filled in is left blank. Our hypothesis is that non-binding defaults can prime subjects and suggest a specific norm of behavior to them. As suggested by Thaler and Sunstein[19], human beings are loss averse, sometimes lazy, and they often also do not pay attention when choosing. For these reasons, a non-binding default option can effectively help them to make better decisions without restricting their choices.Classical examples of effective defaults are the automatic enrolment in retirement saving [20,21] or the opt-out vs. opt-in organ donation scheme [22].

The previous literature on this topic has focused on the effect of default on the cooperation decision. For instance, Altmann and Falk [23] show that the use of default options in a public goods game mainly influenced the behavior of people with low cognitive skills. In addition, Cappelletti et al. [24] show that preferences for a suggested contribution significantly increase when it is presented as the default, while Liu and Riyanto [25] tested the effect of Opt-In and Opt-Out options on the level of contribution. However, all of these papers look at the effect of different defaults on individual contributions and less on the effect that a default may have on priming participants towards different norms of behavior. This type of analysis is more in line with the recent study by Drouvelis et al. [26] who found that cooperative priming increases contributions in a one-shot public goods game by around 45% compared to the non-primed group. However, the authors did not study the effect of this priming over time or when the default is not present anymore.

To guide our investigation of this experiment, we suggest the following hypotheses: *First*, we anticipate that the default answer, that we implement, will be used by the subjects. In line with the existing evidence of defaults[20–22], we expect that providing our subjects with an answer which is easy to accept, i.e. the default, will tempt them to select this option. *Second*, we consequently also expect that the classification of the subjects’ types will be affected by the manipulation. In particular, we expect more free rider preferences in the FR treatment and more perfect conditional cooperators in the PCC treatment. Third, we also expect that the default in the PCC treatment will generally influence preferences to be more cooperative (besides increasing the number of perfect conditional cooperators). And *fourth*, we suggest that the manipulation in PCC puts subjects into a particular mental state, which leads them to anticipate certain social norms, which then influences their behavior in the subsequence repeated public goods game (where a default is not used). In other words, we expect that subjects contribute significantly more in PCC compared to both the ND and FR treatments.

## Materials and Methods

**Fig 1:**
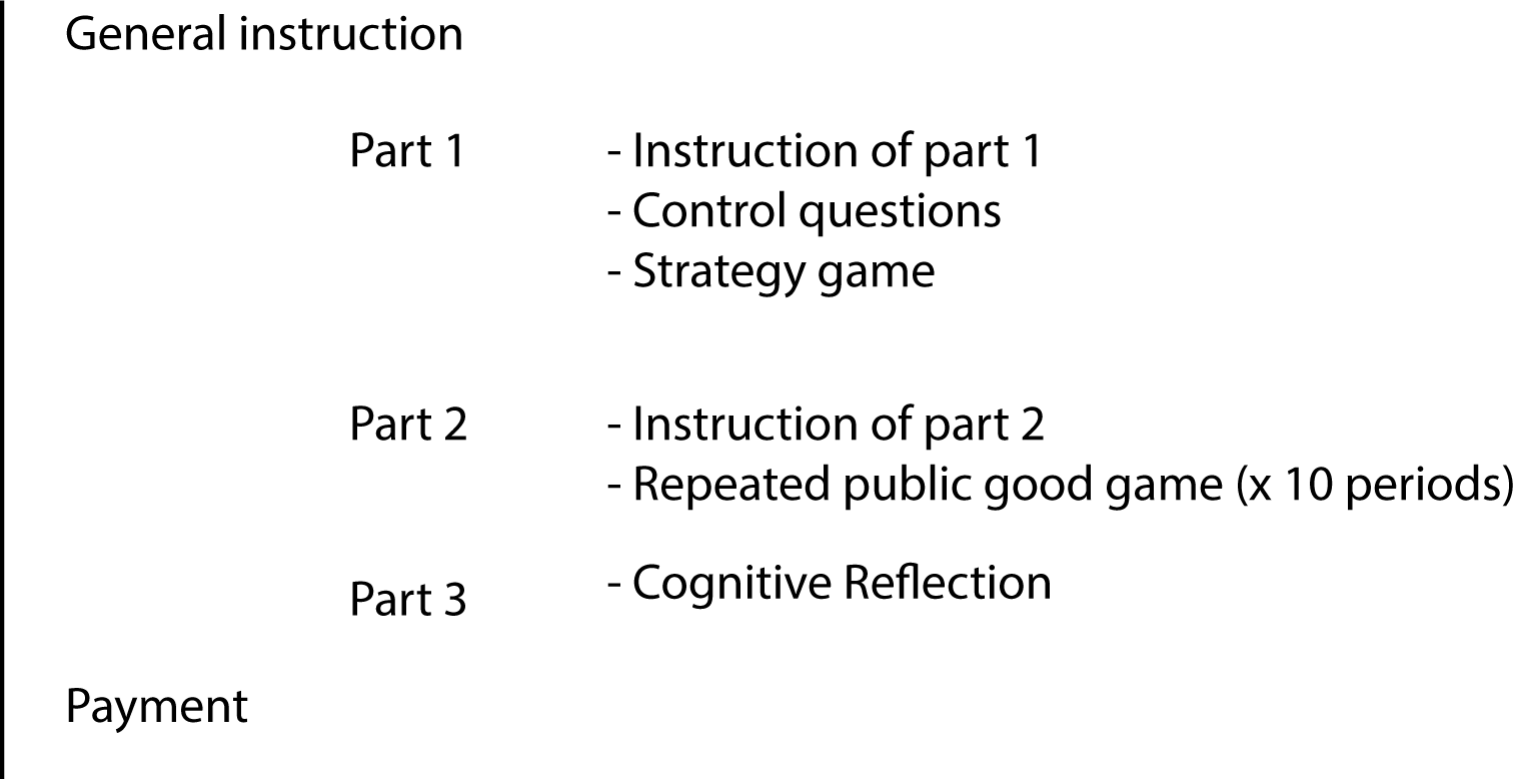
Timeline of our experiment.

The experiment was conducted at the Laboratory for Experimental Economics (LEE) of the University of Copenhagen. A total of 227 first-year undergraduate economics students participated, aged from 18 to 36 (mean 20.5, sd 1.9). Subjects were entirely anonymous and no indication of their identity was collected during this study. The subjects had not participated in a public good experiment before. Upon arrival, the subjects were seated in individual booths and received written instructions about the public goods game. Then we informed the subjects that the experiment consisted of two parts and a final questionnaire. We asked control questions to ensure full comprehension before each part. The Supplementary Information reports the complete set of instructions, the control questions, and selected screenshots (S1 File). Fig 1 summarizes the timing of our experimental design with the exact sequence of tasks and decisions.

### Strategy game–Part 1

In part 1, subjects play a one-shot public goods game in groups of 4. Each subject is initially endowed with 20 points and has to choose how many of their private points to put into a public pot. The points in this pot are multiplied by two and this "public good" payoff is evenly divided among the four subjects. Each subject also keeps the points they do not contribute.

Following the protocol of Fischbacher et al. [11], we asked subjects; 1) how many points they wanted to put in the public pot and; 2) their profile of conditional contributions, i.e., their contribution conditional for all the possible average contributions of the other group members (that is from 0 to 20). Thus, subjects have to indicate 21 conditional contributions (21 numbers in 21 cells), one for each possible (integer) average contribution of the other group members. As explained in the subjects’ instructions, to calculate their payoffs for Part 1, we used the elicited unconditional contributions for three randomly selected group members, while the fourth subject’s contribution was calculated based on the elicited conditional contribution profile using the average of the unconditional contributions from the other three group members.

We vary the conditional contribution elicitation across three treatments in the following way: In the No Default Treatment the cells to be filled in were left blank. In the Free Rider Treatment the default in each cell is a “0” whereas in the Perfect Conditional Cooperators Treatment the default in each cell corresponds to the average contribution of the other group members (0,1,2,…,19,20). It is important to notice that, in FR and PCC, all the default numbers could be erased and a new number could be typed in.

In total, we have 9 sessions (three per treatment) with 46 subjects in ND, 105 subjects in FR and 76 in PCC treatment The number of participants is not dividable with four (which is the number of subjects in each group in the public good game). The reason is that we allowed for not complete groups in order not to send home any of the subjects. In practice this is implemented by filling up the not complete groups with ghost members who are members from other complete groups, and reuse their decisions. This procedure implies that all participants experienced to be in a complete group, and the matched decisions were real in the sense that they reflected actual decisions of other subjects within the same session. The participants were not informed about this. 15 out of the 227 participants were in this situation (6.6%). At the end of this part we do not provide subjects with any feedback, but they know feedback will be provided at the very end of the experiment. The SI contains the complete set of instructions and some selected screen shots.

### Repeated Public Good Game–Part 2

At the beginning of Part 2 we distribute a new set of instructions. In Part 2 subjects participated in a repeated public good game where the basic parameters are exactly the same as in Part 1: the initial endowment is 20 points, and public good contributions are doubled and squared equally. The game was repeated for 10 periods: after each period, feedback about other group members’ average contribution and own income was provided and the computer create new groups of four subjects (*stranger matching* protocol).

### Cognitive Reflection Task

Upon completion of the repeated public good game, we asked subjects to do the Cognitive Reflection Test (CRT) [27]. The test consists of the following three questions:

1. A bat and a ball cost $1.10 in total. The bat costs $1.00 more than the ball. How much does the ball cost?
2. If it takes 5 machines 5 minutes to make 5 widgets, how long would it take 100 machines to make 100 widgets?
3. In a lake, there is a patch of lily pads. Every day, the patch doubles in size. If it takes 48 days for the patch to cover the entire lake, how long would it take for the patch to cover half of the lake?

All these three questions have an immediate and intuitive (but incorrect) answer and a more cognitively demanding (but correct) answer. The test measures whether a subject tends to give fast intuitive answers, rather than carefully trying to derive the correct answer. The more correct, as opposed to immediate and intuitive, answers a subject gives, the more cognitively reflective she is.

### Ethics Statement and data availability

In accordance with Danish legislation [28,29] there was no need for an institutional review board approval (IRB) for this study, as sensitive data—as defined by the Danish Data Protection Agency—was not retrieved from participants. The study was, however, revised and approved by the Center for Experimental Economics at the University of Copenhagen which reviewed the process to ensure that the consent procedures, instructions, and data collection were conducted in line with good ethical research practice. Our participants volunteered to participate and show up for the study. Subjects were introduced to the topic of the experiment on the first page of the instructions and could withdraw, at any time, if they did not want to participate. Participants therefore provided their verbal informed consent to participate in the study. No written consent was obtained as it is not required by the rules of the Center for Experimental Economics at the University of Copenhagen. The participants signed a certificate of participation at completion of the experiment when paid. The certificate formally acknowledges their participation. All participants signed the certificate. The complete dataset, without any identification of the participants, is posted on Figshare.

## Results

In this section, we present our four main results.

### Result 1: Subjects do not automatically confirm the default numbers

Our subjects were active in the strategy game. In contrast with our conjecture, most of them delete the default answers which were pre-typed on the screen, and reported another answer. 91.43% of the pre-typed defaults in the FR treatment were deleted, whereas 81.58% of the defaults in the PCC treatment were deleted. The difference in the tendency to accept the default answers across the treatments is marginal significant (Pearson chi square test, chi2(1): 3,856, p = 0.050), but is obviously influenced by the fact that the appeal of the default strategies may vary even without the default manipulation. In other words, it may be the case that fewer people change the default in the PCC treatment than in the FR treatment because more people have preferences for perfect conditional cooperation than for free-riding. Yet, we cannot determine whether those who accept a default do it because she/he is nudged to do so or because she/he actually prefers the answer which happens to be the default.

### Result 2: Different defaults do not affect the classification of subjects into types

We categorize the elicited profiles of conditional contributions into several types in accordance with the standard approach [11]. In Table 1, we list the fraction of each of these types. The most common profile by far is the conditional cooperator type. Across the three treatments, this group accounts for 60,8% of all the subjects.

**Table 1:** The distribution of cooperation preferences across the two default conditions.

	FR Treatment	PCC Treatment	ND treatment
	%	%	%
Conditional Cooperators	60,0	65,8	54,4
Perfect Conditional Cooperators	16,2	**18,4**	13,0
Free Riders	**8,6**	2,6	6,5
Triangle Contributors	9,5	6,6	6,5
Negative Conditional Cooperators	0,0	1,3	0,0
Unconditional Cooperators	1,9	4,0	15,2
Other	3,8	1,3	4,4
Total	100,0	100,0	100,0

Table 1 also contains conditional cooperation profiles which simply accept the two default manipulations. The percentage figures which are in bold are the subjects who confirmed the default per treatment. It can be seen that the FR treatment moves the share of free riders from 2.6% to 8.6% (a difference of 6 percentage points) when comparing PCC and FR, while compared to ND it is an increase from 6.5% to the 8.6% (2.1 percentage points). Comparing FR and PCC, we find that the perfect conditional cooperator defaults raise the share of perfect conditional cooperators from 16.2% to 18.4% (2.2 percentage points). Compared to the ND treatment, the increase is even larger, namely from 13.0% to 18.4% (5.4 percentage points). Thus, many subjects seem to be affected by the default. In the ND treatment, which is parallel to the experimental design of [11], we find a relatively large share of unconditional cooperators. Importantly, this is in itself not a concern but rather an expression of different social preferences. Interestingly, when comparing ND with the default treatments, it is clear that the defaults have an equal effect in reducing the share of unconditional cooperators, and increase the share of conditional cooperators.

By means of a non-parametric Pearson Chi square test, we find that the distribution of cooperation types does not differ across the treatments (chi2(12) = 18.593, p = 0.099). The same conclusion is reached when making the same overall comparison with a Kruskal-Wallis equality-of-populations rank test (Chi2: 0.894, p: 0,640).

Furthermore comparing the FR and the PCC treatments only we also find that the distributions do not differ significantly (chi2(6) = 6.43, p = 0.38) Thus, the initial defaults seemed to affect the subjects’ choices unevenly; however, it turns out that the distribution of types is not significantly different across the treatments, and hence the significant difference observed in the use of the default answers is simply driven by the fact that more people are perfect conditional cooperators than free riders. It should be noted that a pairwise comparison of the distribution of types is found to be marginal significantly different between the ND and FR treatments, suggesting that the FR default analyzed alone might have an effect on the distribution of types, which the PCC defaults analyzed alone did not. Making pairwise comparisons give the following: Fr to PCC chi = 6.43, p = 0.38, PCC to ND chi = 8.29, p = 0.22, FR to ND: chi = 10.42, p = 0.06.

Although the defaults do not affect the overall distribution of cooperative types, it may still affect the subjects’ stated profile. The idea is that even though two subjects are categorized as, for instance, conditional cooperators, they can still be more or less cooperative.

### Result 3: The perfect conditional cooperation default influences subjects to adopt a more cooperative strategy profile

**Fig 2:**
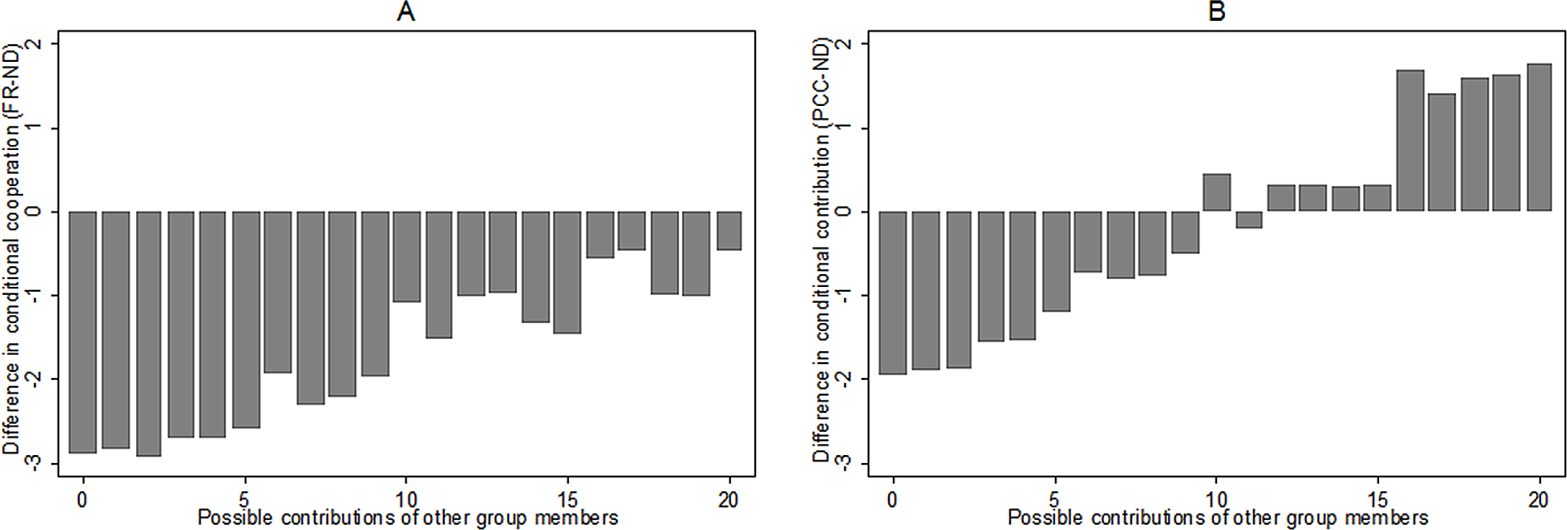
Difference in conditional cooperation answers in the two treatments.

Even if the default does not nudge subjects into accepting the provided default, we do find that subjects’ answers in part 1 are affected by the manipulation. Instead of simply accepting the default answer, subjects deleted it and provided an alternative answer, which was influenced by the default. To illustrate this effect, Fig 2 shows the difference between the average responses in each cell (a certain degree of behavior from other group members) in the conditional table. Fig 2, panel A, shows the average in the PCC treatment minus the average in the ND treatment, whereas panel B shows the average responses in FR treatment minus ND treatment. As is evident from the graph, the average answers in the FR treatment are consistently below those in the ND treatment. That is, the default in FR seems to affect negatively the conditional contributions stated. Whereas, the effect of the default in PCC is asymmetric: for conditional cooperation below 10 the default in PCC decreases contribution compared to ND; for conditional cooperation above 10 the default in PCC increases contribution compared to ND. The PCC defaults are in other words making subjects more conditional in their response to the behavior of other group members.

In fact, across all the subjects, we find that the average answer for all cells of the table is 7.9 in the FR treatment and 9.5 in the PCC treatment. This difference is significantly different (Non-parametric Mann Whitney test, z = -2.693, p = 0.007). Thus, in sum we find that the defaults significantly influence peoples’ reported answers.

Another way the default answer affects our subjects’ behavior is by influencing the subsequent repeated public goods game played in part 2. The idea is that the particular default primes or puts people in a particular mental state, which leads them to view the public good game (slightly) differently.

### Result 4: the effect of the default is persistent even in part 2 (repeated public goods game) where a default is not used

**Fig 3:**
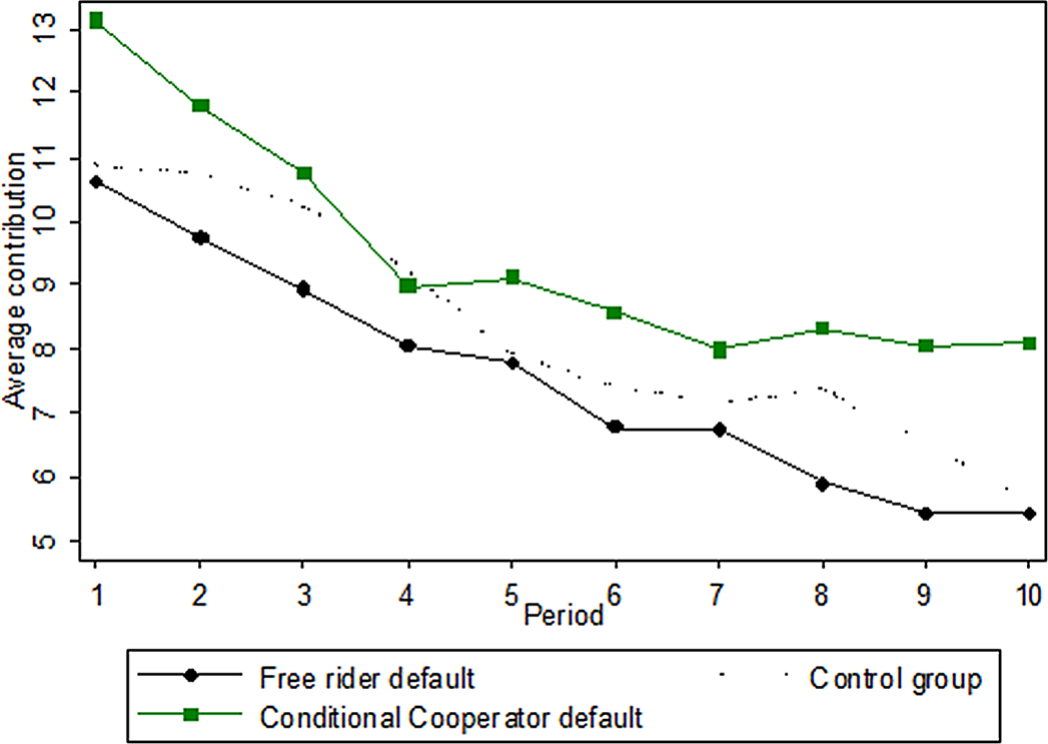
Average public good contribution over periods in the two treatments.

Fig 3 shows the result of the repeated public goods game in the treatments. Over time, the contributions decay, as is typically observed in such games, but interestingly, the average contributions in the PCC treatment are consistently above the contributions in the FR treatment. When evaluating the individual average contributions over all periods across PCC and FR, we find that there is a highly significant difference across these two treatments (Non-parametric Mann Whitney test, z = -2.791, p = 0.005). Note also that the effect does not seem to decline over the repetitions of the game: the difference in mean contribution in the first half (period 1–5) of the repeated public goods game is 1.72, whereas it is 2.14 in the last half of the game (period 6–10). Interestingly, the contributions in the ND treatment are between PCC and FR, suggesting that PCC tend to pull up contributions whereas FR is having the opposite effect.

The effect from our manipulation in Part 1 to the behavior in the repeated public good game could be explained as an anchoring effect. The concept of anchoring is a well-known phenomenon that numbers, or mental states, are carried over from one stage to behavior in a subsequent, and an objectively unrelated, stage. Ariely and co-authors for instance found that stating the last digits in the social security number just before stating willingness to pay for various items had a significant impact on the willingness to pay [30]. In the present experiment the idea is that the particular default numbers, provided in part 1, anchors the participants in the subsequent public good game to state contributions which tend to follows the defaults.

As final step in our analysis, we estimate the effect of the treatments econometrically by applying a random effects GLS model on our panel data. The random effect version was chosen to take account of the fact that the same subject makes 10 repeated decisions.

The regressions are reported in Table 2. In the first regression, we account for the decay effect observed in Fig 3. As expected, the contributions systematically, and significantly, decrease with repetition (see the period variable). Secondly, we add the variable of our main interest, namely the treatments; we have the FR treatment and the ND as dummies and the PCC as a reference group in model 1–5. Importantly we re-confirm that the subjects in the PCC treatment contribute significantly more than those in the FR treatment. We also see that those in the ND treatment do not differ significantly from those the PCC treatment. In model 6, we use the FR treatment as reference group instead, and again we observe the significant difference between the FR and the PCC treatments, but also that the FR treatment does not differ significantly from the ND treatment. Thus, in essence we find that the FR and PCC differ significantly, but they do not differ from the ND treatment which in Fig 3 is located between the two default treatments.

**Table 2:** Panel data random effects generalized least squared regressions.

	(1)	(2)	(3)	(4)	(5)	(6)	(7)
Dependent variable: Contribution							
ND treatment		-1.169	-1.168	-1.169	-1.342	0.766	0.565
		(1.002)	(1.001)	(1.000)	(1.023)	(0.949)	(0.971)
FR treatment		-1.935***	-1.930***	-1.928***	-1.907***		
		(0.738)	(0.736)	(0.739)	(0.731)		
PCC treatment						1.935***	1.907***
						(0.738)	(0.731)
Period	-0.569***	-0.569***	-0.569***	-0.569***	-0.569***	-0.569***	-0.569***
	(0.0418)	(0.0418)	(0.0418)	(0.0419)	(0.0419)	(0.0418)	(0.0418)
Female (0: Male, 1: Female)			0.407	0.421	0.258		0.258
			(0.654)	(0.707)	(0.691)		(0.691)
Cognitive Reflection Score				0.0270	-0.0288		-0.0288
				(0.348)	(0.344)		(0.344)
Age					0.248*		0.248*
					(0.139)		(0.139)
Constant	11.49***	12.62***	12.46***	12.41***	7.500**	10.68***	10.68***
	(0.404)	(0.587)	(0.661)	(0.902)	(2.972)	(0.532)	(0.532)
							
Observations	2,270	2,270	2,270	2,270	2,270	2,270	2,270
Number of id	227	227	227	227	227	227	227
r2 (overall)	0.0570	0.0725	0.0733	0.0734	0.0780	0.0725	0.0780
chi2	185.3	204.8	204.9	205.3	215.1	205	215.1

Robust standard errors in parentheses

*** p<0.01

** p<0.05

* p<0.1

Note: The PCC treatment serve as the references group in model 1–5 whereas the FR treatment is the reference group in model 6.

To test for robustness, we add gender, cognitive reflection, and age as control variables in the model 3–5. We find that neither gender nor cognitive reflection significantly affect contribution behavior, whereas age does. In fact, higher age is highly significantly associated with higher contributions. Interestingly, when controlling for these variables, the effect of the treatment and period remains, which suggests that these effects are robust. In the case of cognitive reflection, this is particularly interesting as [23] found that the effect of the default manipulation (in their case directly in the repeated public good game) was more pronounced among people with less cognitive skills. In the final regression, we again use the FR treatment as the reference group, and we then add the same set of control variables in order to illustrate that the treatment effect in model 6 is also robust to the inclusion of the control variables.

## Conclusion

In this paper, we test the effect of non-binding defaults on cooperation. We manipulate the default numbers that appear on the screen with the elicitation of strategy in the public goods game. In one treatment, the default number in the measure is a free-rider strategy, whereas in another treatment, the default answer corresponds to a perfect conditional cooperator strategy. Our results show that the vast majority of subjects erased the default number and the resulting classification of the measure is the same in the two treatments. However, the answers are still affected by the erased default. Despite the fact that the strategies are classified as being the same, the subjects who saw the perfect conditional cooperator default report significantly more cooperative choices (in the strategy game) than those who saw the free rider default. An even more interesting effect is observed in the subsequent repeated public goods game where there is no default. In this game, we find that the subjects who saw the perfect conditional cooperator default gave significantly more to the public good compared to those exposed to the free rider default. Our findings suggest that cooperation can be fostered (or reduced) by subtle manipulations, and that the ways this effect work are not obvious. The default directly affects the stated amount in the strategy measure, and the default manipulation is so strong that it carries over to the subsequent game, and remains in play throughout this repeated game.

All in all, this evidence suggests that the scope of default manipulations extends way beyond the actual choice in which the default is implemented. It may be possible to use the default to put people into a certain mental state which then influences their decision making. In our study, the subjects are more influenced to adopt an egoistic free rider state-of-mind (in the FR default), while they are more influenced to adopt a cooperative state-of-mind in the PCC default. Such insights may be helpful for steering peoples’ cooperation, and as such our findings contribute to the literature by identifying various cooperation enhancing mechanisms.

## Supporting Information

S1 File Supplementary information. Contains instructions, control questions, and selected screenshots.
(DOCX)
