# Supplementary material for: Nudge for (the Public) Good: How Defaults Can Affect Cooperation
Source: PLoS One. 2015 Dec 30;10(12):e0145488. doi: 10.1371/journal.pone.0145488 (PMC4696855; doi:10.1371/journal.pone.0145488)

# **Supporting Information for:**

# **Nudge for (the Public) Good: How Defaults can affect Cooperation**

## Instructions

Please read the following instruction carefully.

Throughout the entire experiment you can collect points. At the end of the experiment your points will exchanged into kroner with the following exchange rate

**3 points = 1 krone**.

After calculating your earnings in kroner we rounded up the amount to the nearest 5 kroner.

You are going to participate in an experiment with two parts. First we provide you with general information, and then you answer some control questions on the computer to see if you understood these instructions. Thereafter, each part of the experiment will be explained in separate instructions. Instructions for part 1 is on the last pages of this document whereas the instructions for part 2 is in the envelop on your desk. We kindly ask you only to look at the general instruction now, and wait with the instructions for part 1 until the computer instructs you to look at them. Also, please do not open the envelop before part two begins (The computer program will let you when to open the envelop).

**General information**

In this experiment you will be randomly assigned to a group with three other participants. None of you will ever learn who is in the group. The other group members can be all other people in the experiment, including people in the other room.

Every group member received a start amount of **20 points** from us. You, and the other group members, have to decide to keep or to contribute all or some of the points to a common pot, a public good. Every group member is in exactly the same choice situation.

The points you choose to keep are simply yours. The total amount which all group members contribute to the public good is doubled by the computer and shared equally among all four group members. Every group member get the same share of the public good regardless of her/his own contribution.

Here are some examples of how the income is calculated:

Example 1:
Imagine that you and the three other group member each contributes your 20 points to the public good. This means none of you keep anything and the total amount contributed is therefore 80 points. This is doubled to 160 and shared equally among all four group members. Each group member therefore earns:

Start amount – contribution + share of public good: 20-20+40=40 points

Example 2:
Imagine that you and the other group members each contribute 0 points to the public good. This means everyone keep the 20 points. Total contribution is therefore 0 points, nobody receives anything from the public good. Yours and the other group members income is therefore:

Start amount – contribution + share of public good: 20-0+0=20 points

Example 3:
Imagine now that you contribute 15 points while the other group member each contributes 20 points. That is you keep 5 points while the other are not keeping any points. The total contribution is 75 points, which is doubled to 150 points and shared equally among all members, such that each gets 37,5 point from the public good. Your income is:

Start amount – contribution +share of public good: 20-15+37,5=42,5 points,

while the other earns:

Start amount – contribution +share of public good: 20-20+37,5=37,5 points

**Instructions - Part 1**

As explained in the general instruction, you are randomly in a group with three other participants. Each of you gets a start amount of 20 points.

In part 1 you have to decide in *two choice situations*:

Situation 1 is just like the choice explained in the general instruction. That is, you decide how much (0-20 points) to contribute to the public good, without knowing what other contribute.

In situation 2 your task is to indicate how much you will to contribute, given the average contribution of other members to the public good. In a table, you will have to fill out your desired contribution for all possible level of average contributions (full numbers) from the other participants. So in the table your decision is how much you want to contribute, when others average contribute respectively, 0,1,2, …, 18, 19,20 points.

**Note:** The computer will automatically insert some numbers in the table (the exact same numbers for all participants) but you can delete all or some of these numbers and type in another number corresponding to your preferred contribution.

When everyone in the group has decided in both situation 1 and 2, one group member is randomly selected. For the selected group member, situation 2 will count; for the remaining three group members it is situation 1 that counts. When you decide you don’t know if it is you who are selected or not, you should therefore carefully consider decisions in both situations as they might be relevant for you.

Here are some examples that will help you to better understand Part 1:

Example 1**:**
Imagine it is you who are selected. That means it is your table which counts. For the three other group members it is the decision in situation 1 which counts. Imagine the three other group members contributed 0, 10, and 20 points, which mean the average is 10 points. If you in the situation 2 Table decide to contribute 5 points if the other on average contributed 10 points, then the total amount to the public good is 0+10+20+5=35 points. This amount is doubled to 70 points and shared equally among all participants 17,5 points. And in this case you earn:

Start amount – contribution +share of public good: 20-5+17,5=32,5 points

Example 2**:**

Imagine that it is not you who are selected. This mean, that for you and two other group members it is the decision in situation 1 which counts. Imagine your situation 1 decision was 14 points and the two other participants chose 13 and 18 points, on average you each contributes 15 points. If he selected participant in the situation 2 table decided to contribute 17 points when the three other group members on average contribute 15 point, then the total contribution is 14+13+18+17=62. This amount is double to 124 and shared evenly. Which mean each receives 31 points from the public good.

**Note**: the average of the three none selected participant is round to the nearest full number. For instance will an actual average of 13,5 be rounded to 14. The outcome of part 1 will be calculated when you go on in the experiment and shown to you at the end of the experiment.

# **Instructions - Part 2**

In part 2 you will randomly be assigned to a new group with three other group members.

You and the other group members are deciding on a situation perfectly similar to the one explained in the general instruction. That is, you and the three other group members have to decide how much you want to contribute to the public good out of the 20 points you are given as a start amount.

In part 2, this decision is repeated in 10 periods. In every period each group member receives a start amount of 20 points. You will randomly be **assigned to a new group in each of these periods**.

**Note:** the same rules as the general instruction apply. In every period, the total contribution to the public good is doubled and shared evenly with all four group members. After each decision, and before the next period starts, you will learn how much the three other group members on average contributed.

## Selected screenshots

Figure A in S1 File: Control questions (part 1) #1:


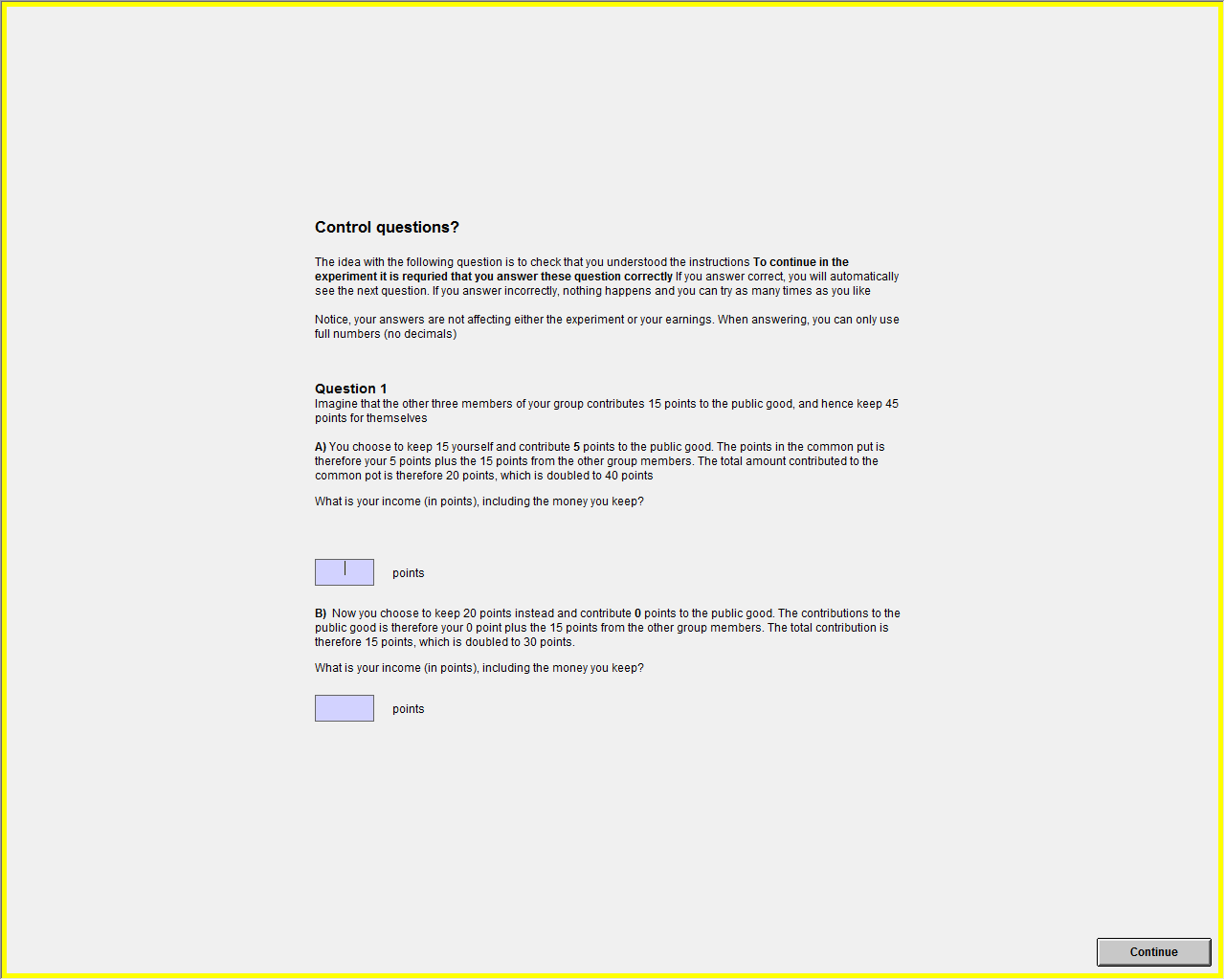


Figure B in S1 File: Control questions (part 1)#2


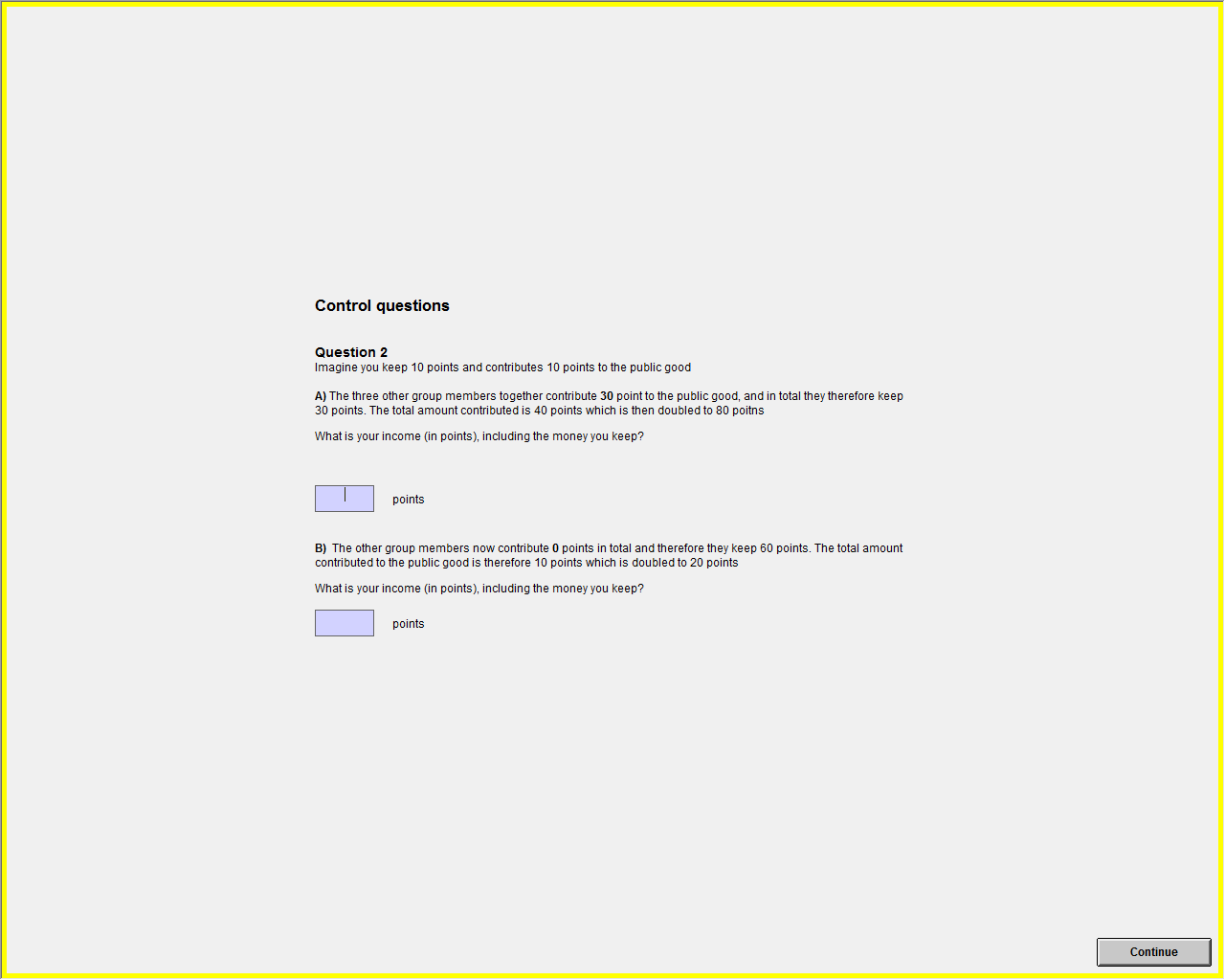


Figure C in S1 File: Strategy game – unconditional choice:


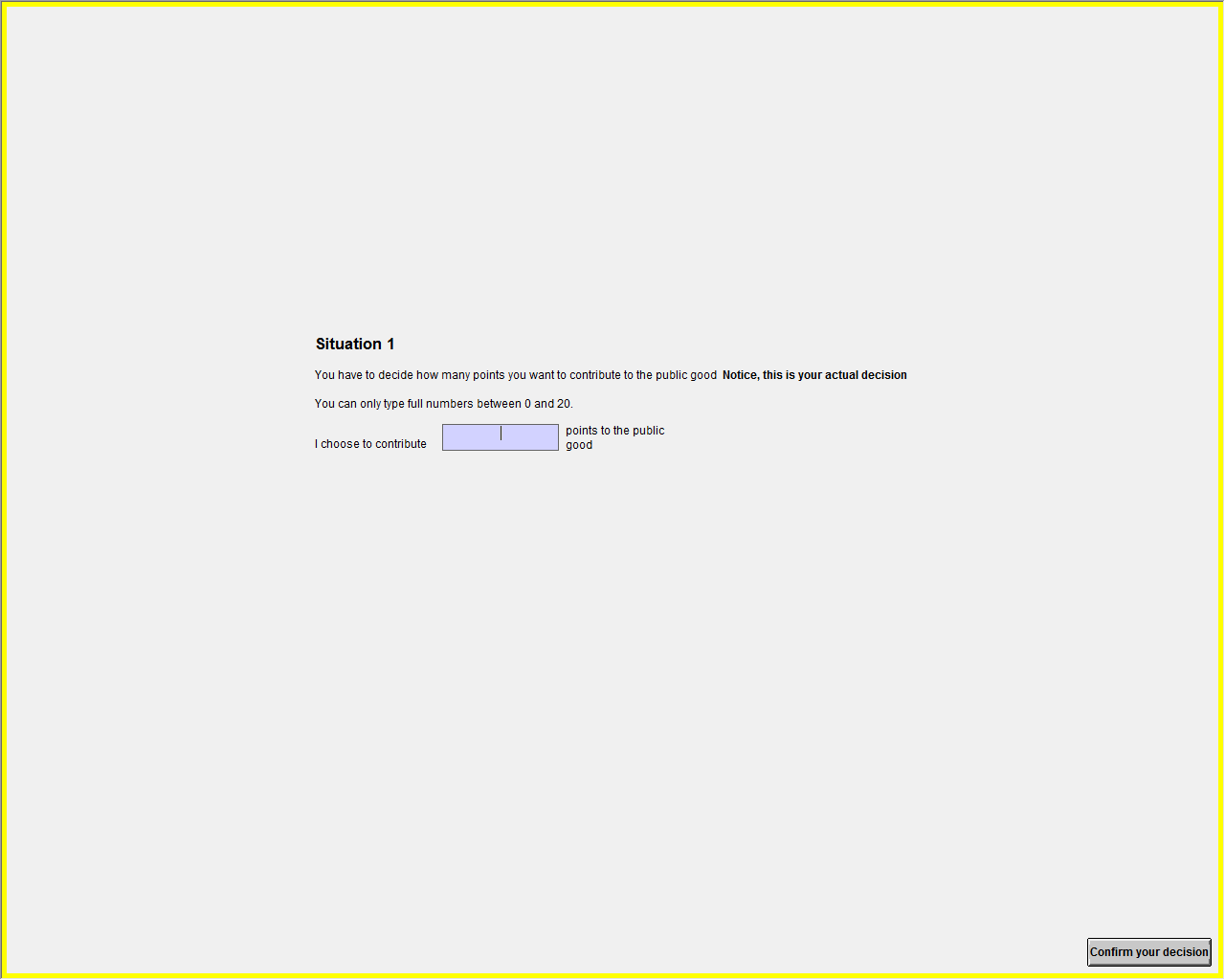


Figure D in S1 File: Strategy game – conditional choices (Here with the free rider default)


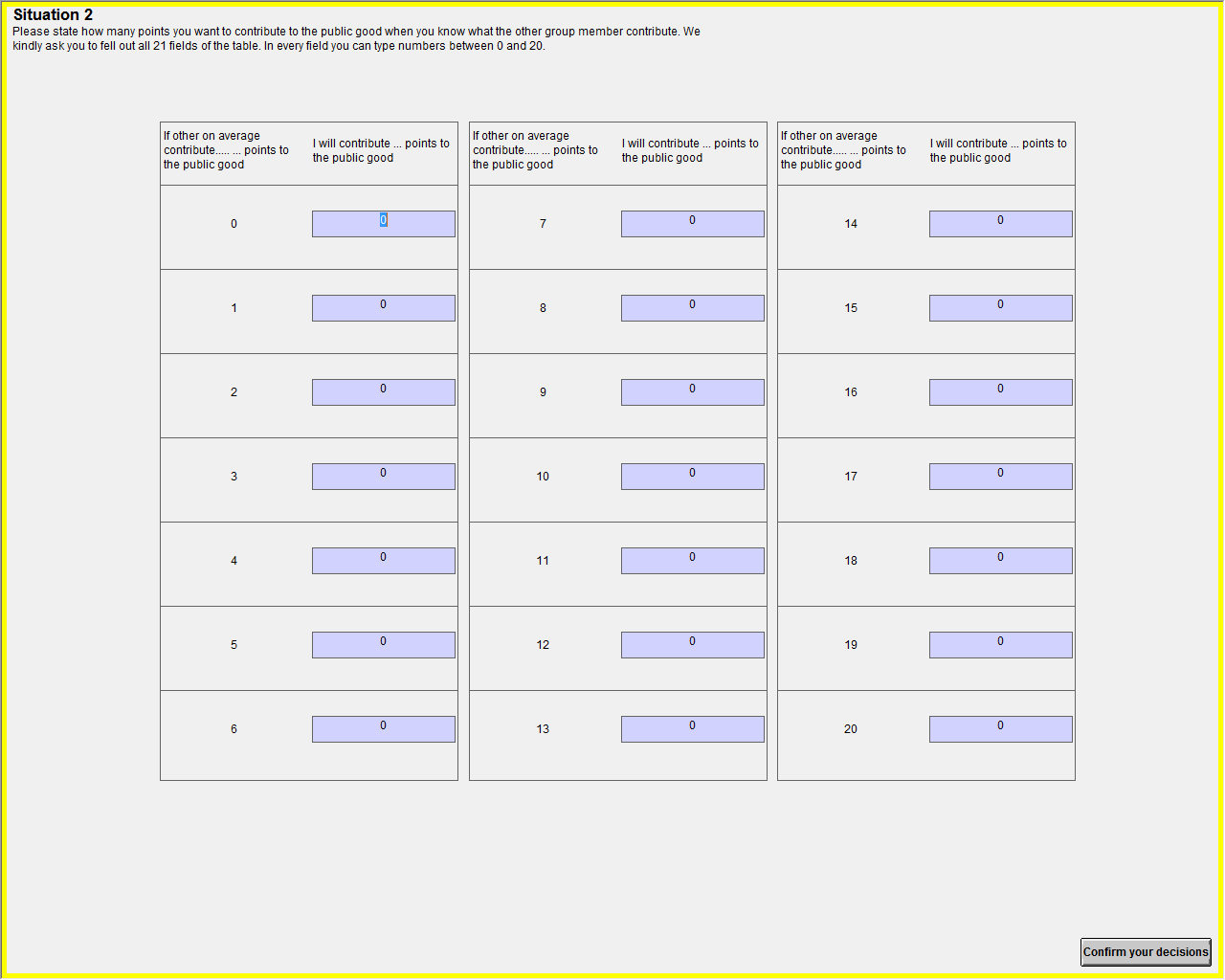


Figure E in S1 File: Control questions (part 2)


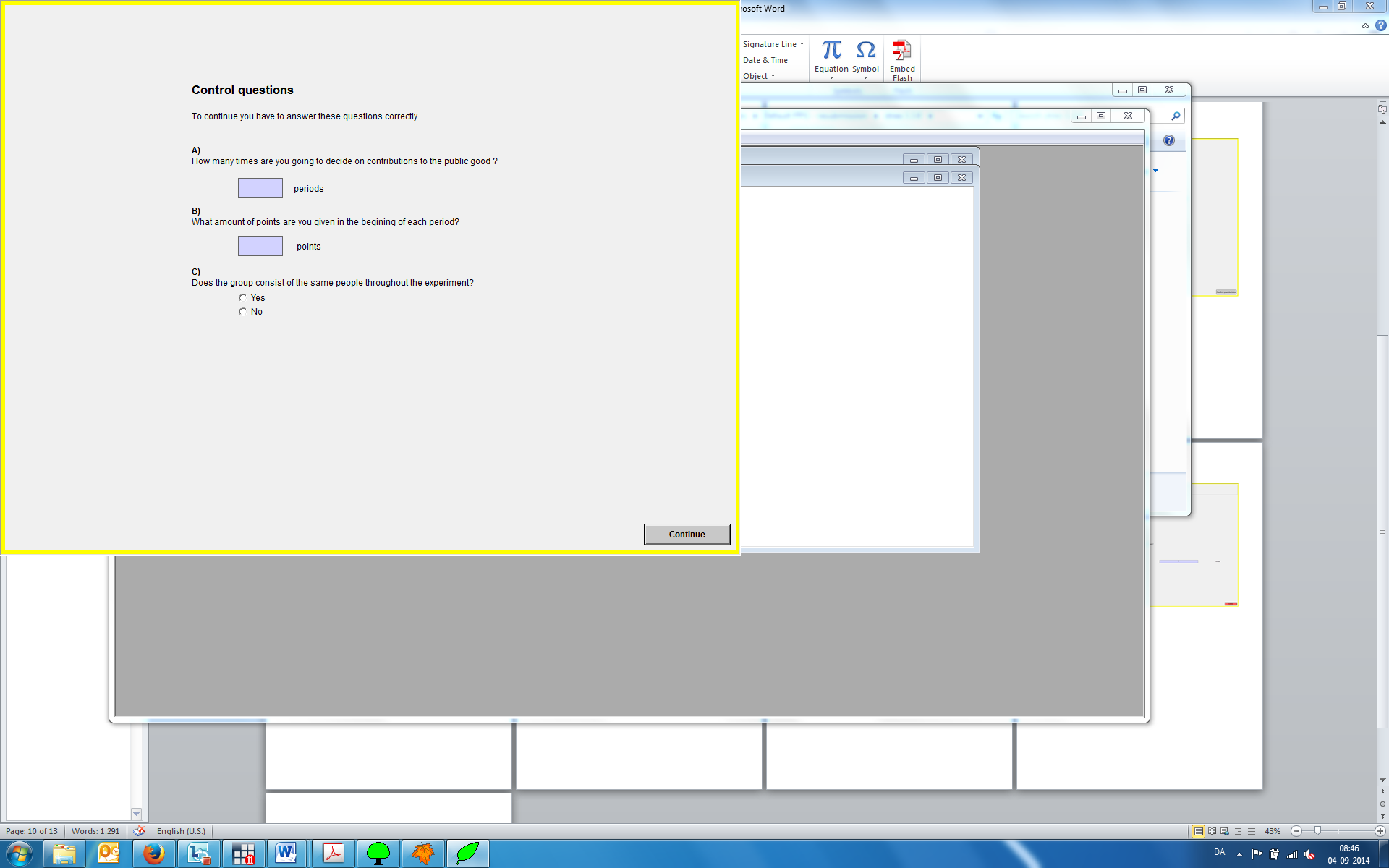


Figure F in S1 File: Repeated public good game
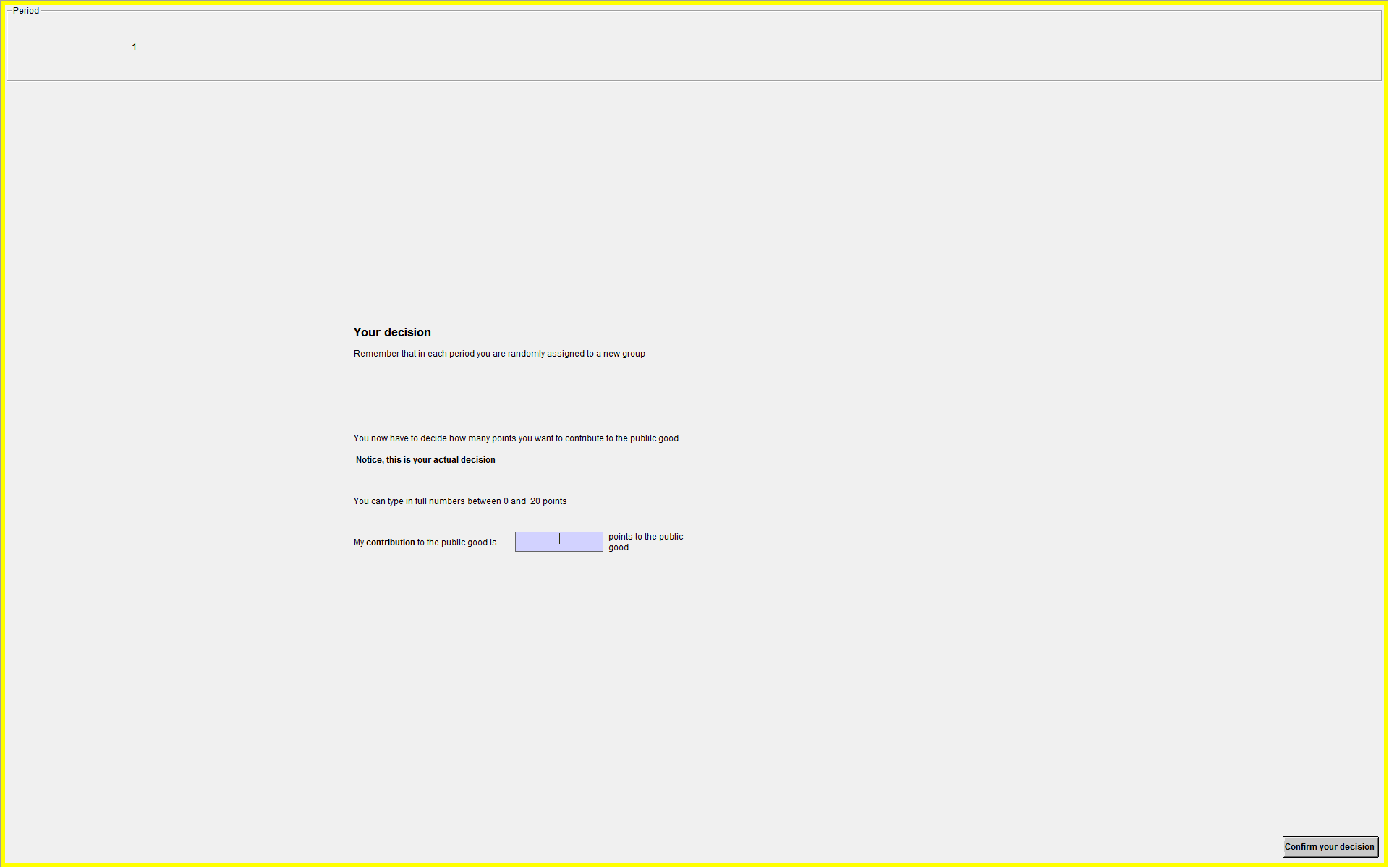


Figure G in S1 File: Cognitive reflection (Question 1)


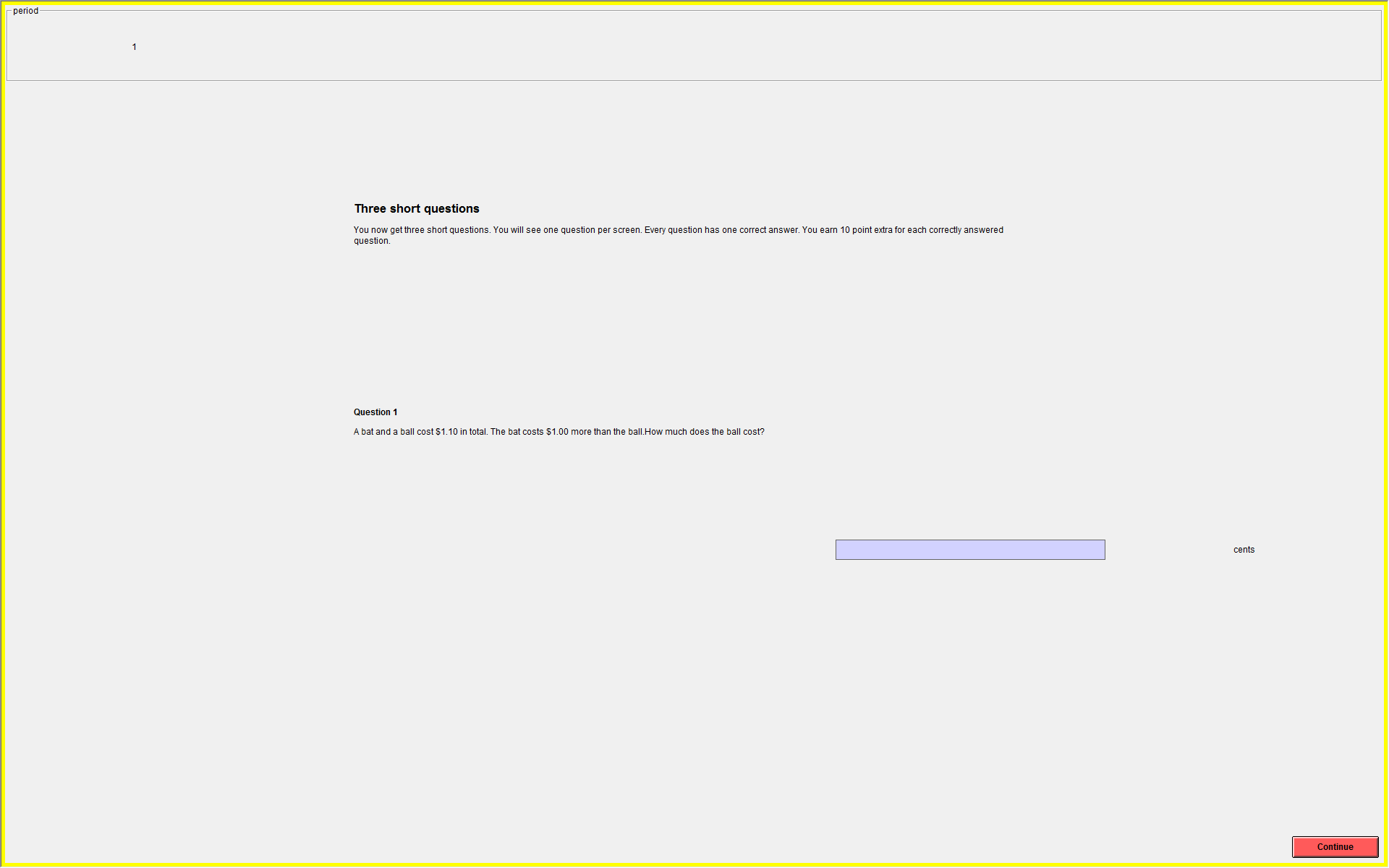


Figure H in S1 File: Cognitive Reflection (question 2)


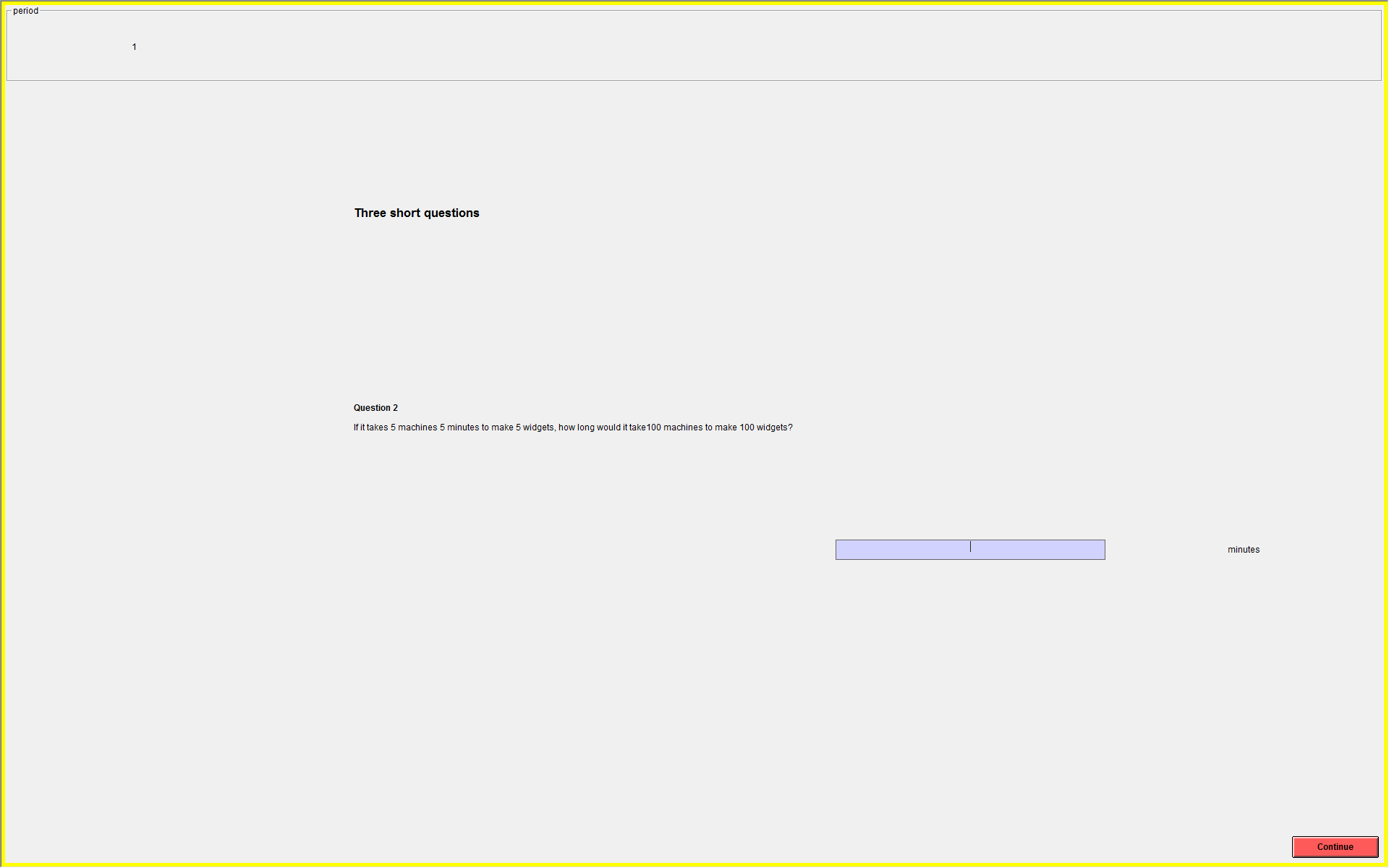


Figure I in S1 File: Cognitive reflection (question 3)


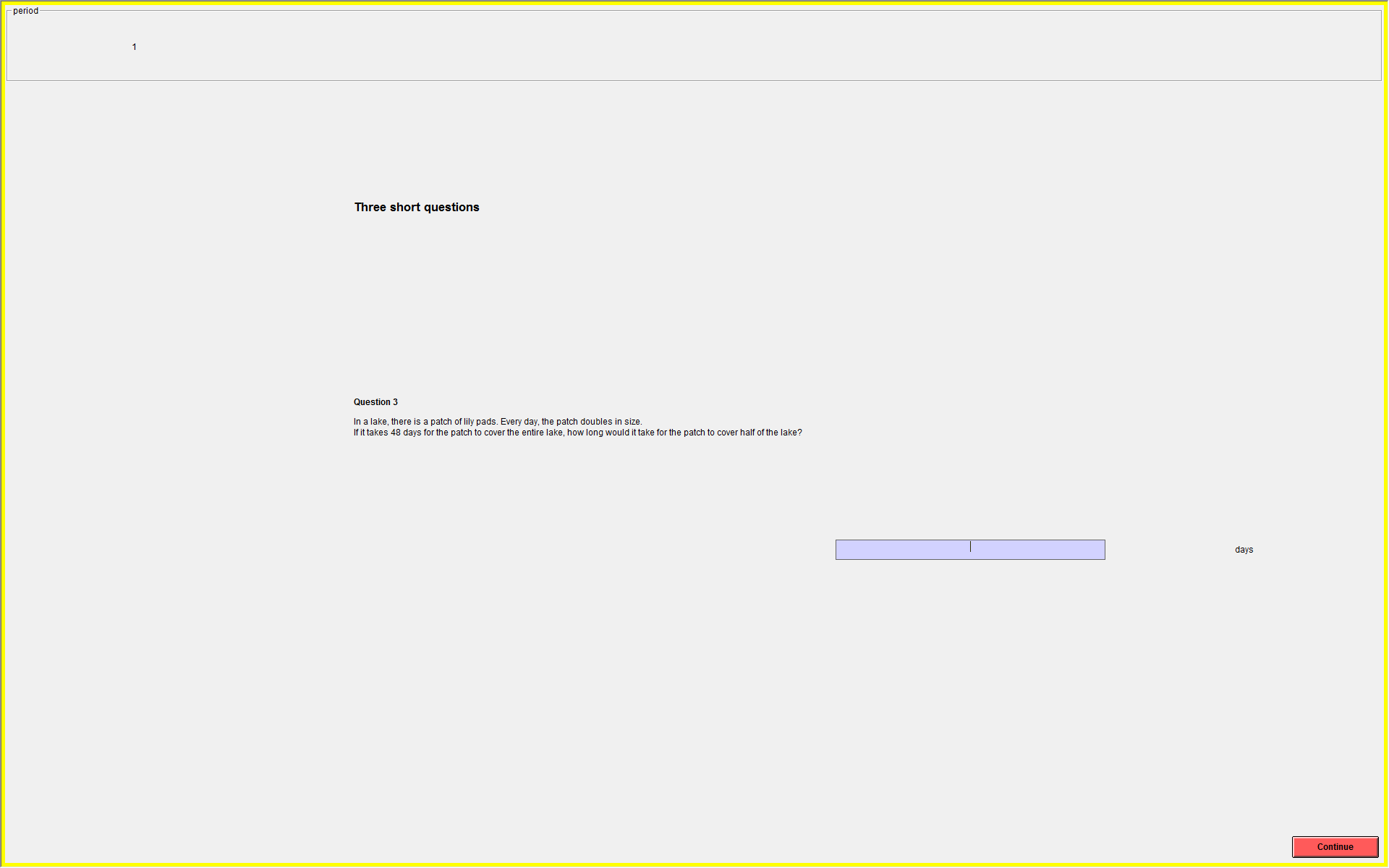

Supplement: S1 File — Contains instructions, control questions, and selected screenshots. (DOCX) [file pone.0145488.s001.docx]
